# Reproductive Immunology Research: A Tight Interaction between Diverse Scientific and Clinical Disciplines Including Immunology, Obstetrics, Hematology, and Endocrinology

**DOI:** 10.3389/fimmu.2015.00010

**Published:** 2015-01-23

**Authors:** Stavros Giaglis, Sinuhe Hahn

**Affiliations:** ^1^Department of Biomedicine, University Hospital Basel, Basel, Switzerland; ^2^Department of Rheumatology, Kantonsspital Aarau, Aarau, Switzerland

**Keywords:** reproductive immunology, immunology, obstetrics, hematology, endocrinology

The unique immunological riddle of human gestation involves a broad variety of cells and molecules playing evidently important roles (1). The reproductive and immune systems are morphologically diffused, and their *modus operandi* involves widely distributed signals in response to the given environmental input, while both systems must interact to obtain their normal functionality (2). Furthermore, dysregulation of physiological interactions between the two systems can lead to severe pregnancy-related disorders or complications, such as fetal loss, preterm labor, preeclampsia (PE), and poor fetal development (3). On the other hand, by ameliorating autoimmune conditions such as multiple sclerosis and rheumatoid arthritis, while other conditions such as systemic lupus are deteriorated, pregnancy as a condition may provide a unique insight into novel immunomodulatory strategies (4, 5).

The scientific focus on reproductive–immune research has historically provided substantial understanding of the interface between these two physiological systems. With such recent progress in the field, we felt that it is valuable and well-timed to review the broad variety of the relevant physiologic and pathologic aspects – from menstruation to fertilization and implantation, and from placentation and pregnancy *per se* to the post partum condition – in which the immune system takes part.

As editors, we are delighted by the keen response of 15 groups of scientists from 4 different continents, which kindly contributed their unique expertise to our effort to recap, value, and extend our insights concerning the field of reproductive immunology. We sincerely hope that the present eBook will succeed to share with the reader the broad and vivid discussion between the authors and editors.

Schumacher et al. (6) explore the interplay between the endocrine and immune systems during gestation, with focus on progesterone, estradiol, and human chorionic gonadotropin. Pregnancy hormones are critically for the successful establishment, maintenance, and completion of pregnancy. They suppress detrimental maternal alloresponses while promoting tolerance pathways through the antigen-presenting capacity of DCs, monocytes, and macrophages as well as the blockage of NK, T, and B cells. These findings highlight the importance of endocrine factors for tolerance induction during pregnancy.

Hsu and Nanan (7) discuss the recent advances in the complex crosstalk between the innate and adaptive immune system during human pregnancy and PE. They present many lines of evidence supporting an immunological origin to PE, implicating decidual NK cells and APC DCs and macrophages as major players in the regulation of vascular remodeling and trophoblast invasion. On the other hand, within the adaptive immune system, Foxp3^+^ Tregs and CD4^+^HLA-G^+^ suppressor T-cells seem to be essential for guaranteeing immune tolerance.

Getting deeper into the mechanisms of placental pathology, Faas et al. (8) focus on the role of monocytes and macrophages in pregnancy and PE. Given the generalized activation of the acute inflammatory response, monocytes may play a central role in this reaction, since they are short-lived cells that mature in the circulation and transmigrate into affected tissues upon an inflammatory stimulus, developing into macrophages. Macrophages in turn are abundant in the endometrium and play a crucial part in implantation and placentation. In PE, these macrophages appear to be activated and in larger numbers.

In the same context, Ruocco et al. (9) provide insight concerning the role of Tregs in pregnancy reassessing the original concept of “suppressor T-cells” in pregnancy, putting it in a historical perspective, and highlighting the main data revising the concept of Tregs in gestation. Moreover, they focus to the most important questions in the field, such as Treg antigen specificity, Treg subsets, the functional crosstalk of Tregs with NK and DCs.

Joerger-Messerli et al. in Basel (10) observed that the inflammatory reaction in monocytes is initiated by the interaction of syncytiotrophoblast microparticles (STBM) with TLRs, which in turn signal through NF-κB to mediate the transcription of pro-inflammatory mediators. Since pregnancy is accompanied by a mild systemic inflammatory response, they show that *in vitro* generated STBM from normal placentas stimulate monocytes. Furthermore, STBM derived from PE placentas up-regulated CD54 expression, and stimulated IL-6 and IL-8 secretion in a dose-dependent manner, which was impaired in the presence of TLR signaling inhibitors or when blocking NF-κB activation.

Fettke et al. (11) examine B cell involvement in the immune response against paternal antigens and tolerance mechanisms. Such pleiotropic cells play a considerable role by secreting immunomodulatory IL-10, while they can harm pregnancy due to their capacity to produce autoantibodies. New evidence in mouse models suggests that IL-10 producing B cells (B10), contribute in maintaining tolerance, fighting danger signals at the fetal–maternal interface. In human pregnancies, B10 cells increase with onset but not in case of spontaneous abortions, suppressing TNF-α production by T-cells.

A review on the unique neonatal NK cell population and its role in gestational autoimmunity is provided by Rival et al. (12) from Kenneth Tung’s group. Maternal autoantibodies can trigger autoimmune ovarian disease (AOD) in the progeny of women with SLE or Sjogren’s syndrome. The pathogenic effect of autoantibody exposure is investigated in mice, in which immune complexes are formed in adult and neonatal ovaries, but a specific Ab-species triggers severe AOD only in young mice. Propensity to AOD is due to the uniquely hyper-responsive neonatal NK cells. Resistance to AOD in older mice results from specific NK cells that regulate effector NK cells and Tregs. Activated by ovarian immune complexes, NKs migrate to lymphoid organs where priming occurs. These insights uncover new properties of the neonatal innate and adaptive responses, lethality of premature infant infection, and novel neonatal antiviral vaccine design.

Woidacki et al. (13) summarize the existing knowledge concerning the course of pregnancy in women affected by mast cell (MC) mediated or associated disorders. While physiological numbers of MCs influence positively the outcome of pregnancy, uncontrolled augmentations in quantity and activation can lead to dangerous complications. Women with the desire of getting pregnant and diagnosed with MC mediated disorders – urticaria, mastocytosis, or MC-related chronic inflammatory diseases – may benefit from specialized medical support to ensure a positive pregnancy outcome.

Than et al. (14) provide evidence concerning the protective role of placental protein 13 (PP13), an immunoregulatory galectin. Three of the five human galectins are expressed in the placenta, and galectin-13 (PP13) is predominantly expressed by the syncytiotrophoblast and released from the placenta into the circulation. Its ability to induce apoptosis of T-cells *in vitro* and to kill T-cells and macrophages in the maternal decidua, suggests important immune functions. Indeed, *LGALS13* mutations and decreased placental expression of PP13 and its low concentrations during first trimester are associated with elevated risk of PE. PP13 turned to be a good early biomarker to assess maternal risk for the subsequent development of pregnancy complications, which might enable its potential in directing patient management.

Sedlmayr et al. (15) highlight the role of tryptophan catabolism in the placenta, focusing mainly on the role of indoleamine 2,3-dioxygenase-1 (IDO1), one of three enzymes involved in the tryptophan degradation pathway. IDO1 has been implicated in regulation of feto-maternal tolerance in the mouse. Depletion of tryptophan mediates immunoregulation and antimicrobial functions. In addition to the decidual glandular epithelium, IDO1 is localized in the vascular endothelium of the chorion and the endothelium of the decidual spiral arteries. Possible consequences of tryptophan catabolism in the endothelium are relaxation of the placental vasotonus, contributing to placental perfusion and growth of both placenta and fetus.

Chatterjee et al. (16) from the Mitchell lab examine how immune cells that produce IL-4 and IL-10 are regulated throughout pregnancy and the effects of reduced IL-4 and IL-10 signaling on fetal and maternal physiology. The resolution of inflammation plays an important role throughout pregnancy and is largely mediated by immune cells producing IL-4 and IL-10. The temporal and spatial aspects of reducing inflammation during pregnancy are thoroughly discussed.

Perez-Sepulveda et al. (17) discuss the involvement of the innate immune system in the establishment of an environment that favors pregnancy and possible alterations related to the development of PE. Since normal pregnancy is considered as a Th2 immunological state, PE has been classically described as a Th1/Th2 imbalance; recent studies have expanded the Th1/Th2 into a Th1/Th2/Th17 and regulatory T-cells paradigm and where DCs could have a crucial role.

An insight into the impact of bacterial infections, such as *Helicobacter pylori* (HP), in PE is delivered by Tersigni et al. (18). Since the primary trigger of PE is unknown, a hypothesis concerning the disease onset is triggering by infectious agents. Consistently, higher seroprevalence of HP infection is evident in women with PE. As trophoblast invasion is a crucial step in implantation and placental development, the proposed infection-induced autoimmune mechanism, interfering negatively with the fetal side of the developing placenta, may explain the higher seropositivity for HP infection PE cases.

Nilsson et al. (19) discuss the role of the human HLA-Ib protein (HLA-G) in the regulation of the immunological crosstalk during conception and pregnancy: from genetics to physiological effects, from pregnancy and pregnancy complications to a short discussion on future possible means of preventative measures and therapies. As HLA-G expression is limited to gestation, it is proposed as a key player in the maintenance of immunological tolerance. HLA-G might be involved in immune processes even before conception, since HLA-G is detected in non-pregnant women genital tract and blood, in men’s seminal fluid, and in the pre-implanted embryo. Therefore, a combined contribution from the mother, the father, and the embryo/fetus is important.

The series of articles included in the present collection is completed with a commentary by Khoury et al. (20) on the promising potential of menstrual stem cells for antenatal diagnosis and cell therapy. Menstrual-derived stem cells (MenSCs) are a new source of MSC isolated from the menstrual fluid. Currently, there is a growing interest in their clinical potential due to their multipotency, high proliferative capacity, and facile way to obtain non-invasively. This review details their distinctive biological properties regarding immunophenotype and function, proliferation/differentiation potential, and paracrine effects. Their possible role in antenatal diagnosis is also discussed.

## Conflict of Interest Statement

The authors declare that the research was conducted in the absence of any commercial or financial relationships that could be construed as a potential conflict of interest.
